# Macrophages Demonstrate Guanylate-Binding Protein-Dependent and Bacterial Strain-Dependent Responses to *Francisella tularensis*

**DOI:** 10.3389/fcimb.2021.784101

**Published:** 2021-12-24

**Authors:** Nasibeh Mohammadi, Helena Lindgren, Masahiro Yamamoto, Amandine Martin, Thomas Henry, Anders Sjöstedt

**Affiliations:** ^1^ Department of Clinical Microbiology and Laboratory for Molecular Infection Medicine Sweden (MIMS), Umeå University, Umeå, Sweden; ^2^ Department of Immunoparasitology, Research Institute for Microbial Diseases, Osaka, Japan; ^3^ Inserm, U1111, Centre International de Recherche en Infectiologie, Lyon, France

**Keywords:** *Francisella tularensis*, Guanylate-binding proteins, macrophages, cytokine patterns, co-infection

## Abstract

*Francisella tularensis* is a facultative intracellular bacterium and the etiological agent of tularemia, a zoonotic disease. Infection of monocytic cells by *F. tularensis* can be controlled after activation with IFN-γ; however, the molecular mechanisms whereby the control is executed are incompletely understood. Recently, a key role has been attributed to the Guanylate-binding proteins (GBPs), interferon-inducible proteins involved in the cell-specific immunity against various intracellular pathogens. Here, we assessed the responses of bone marrow-derived murine macrophages (BMDM) and GBP-deficient BMDM to *F. tularensis* strains of variable virulence; the highly virulent SCHU S4 strain, the human live vaccine strain (LVS), or the widely used surrogate for *F. tularensis*, the low virulent *F. novicida*. Each of the strains multiplied rapidly in BMDM, but after addition of IFN-γ, significant GBP-dependent control of infection was observed for the LVS and *F. novicida* strains, whereas there was no control of the SCHU S4 infection. However, no differences in GBP transcription or translation were observed in the infected cell cultures. During co-infection with *F. novicida* and SCHU S4, significant control of both strains was observed. Patterns of 18 cytokines were very distinct between infected cell cultures and high levels were observed for almost all cytokines in *F. novicida*-infected cultures and very low levels in SCHU S4-infected cultures, whereas levels in co-infected cultures for a majority of cytokines showed intermediate levels, or levels similar to those of *F. novicida*-infected cultures. We conclude that the control of BMDM infection with *F. tularensis* LVS or *F. novicida* is GBP-dependent, whereas SCHU S4 was only controlled during co-infection. Since expression of GBP was similar regardless of infecting agent, the findings imply that SCHU S4 has an inherent ability to evade the GBP-dependent anti-bacterial mechanisms.

## Introduction

*Francisella tularensis* is a highly virulent bacterium and the etiological agent of the zoonotic infection tularemia, a febrile disease affecting many mammalian species, including humans (Sjöstedt, 2007). *F. tularensis* infects hosts *via* a variety of routes, most commonly through arthropod bites, such as mosquito and tick bites, but also through inhalation or ingestion (Tärnvik and Berglund, 2003). Clinical isolates are highly contagious and also demonstrate high, albeit variable, virulence (Molins et al., 2010). The highest virulence is exhibited by strains belonging to subspecies *tularensis* and they are confined to North America, whereas strains of subspecies *holarctica* have been isolated from many locations across the Northern hemisphere and exhibit lower virulence (Kingry and Petersen, 2014). Experimentally, often strains of lower virulence are used and one such example is the live vaccine strain (LVS) belonging to subspecies *holarctica*, an attenuated strain used for vaccination of humans (Conlan, 2011). Also, *F. novicida* is an often used surrogate for *F. tularensis*, since it is genetically very closely related and even has been suggested to be designated as a subspecies (Kingry and Petersen, 2014). However, *F. novicida* is a very rare human pathogen and, compared to *F. tularensis* strains, demonstrates distinct biological features upon intracellular infection; most notably strong pro-inflammatory properties (Cowley and Elkins, 2011; Gillette et al., 2014).

*F. tularensis* is a facultative intracellular pathogen and central to its pathogenicity appears to be the ability to use monocytic cells as the primary replication site (Celli and Zahrt, 2013). Upon its encounter with a host cell, a proinflammatory response occurs, which is repressed when bacterial internalization has occurred (Telepnev et al., 2005; Dai et al., 2013; Bauler et al., 2014; Scott et al., 2017; Jessop et al., 2018). The muted inflammatory response is concomitant with the escape of *F. tularensis* from the phagosome into the cytosol (Celli and Zahrt, 2013). Upon entering the cytosol, rapid intracellular replication ensues. In addition to the muted inflammatory responses during internalization, *F. tularensis* also suppresses the inflammatory responses of various types of monocytic cells to secondary stimuli (Telepnev et al., 2003; Schwartz et al., 2012; Ireland et al., 2013). Thus, central to the interaction with the monocytic cell is the ability of the bacterium to modulate the host inflammatory response and this is essential to its pathogenicity.

Previous findings have demonstrated that host cells display very distinct responses to different strains of *F. tularensis*. For example, *F. novicida* is not only used as a surrogate for virulent *F. tularensis*, but also as a prototypic organism for investigations of the AIM2 inflammasome (Fernandes-Alnemri et al., 2010; Wallet et al., 2016; Wallet et al., 2017, Zhu et al., 2018a, Zhu et al., 2018b). Ingestion by macrophages of *F. novicida* leads to a TLR2-dependent, pro-inflammatory response and upon the phagosomal escape, recruitment of GBPs to the cytosolic bacteria, leading to lysis and release of genomic DNA (Wallet et al., 2016). This cytosolic DNA is then recognized by AIM2. Activation of the AIM2 inflammasome leads to caspase-1 activation and this is a prerequisite for the Gasdermin D-dependent secretion of the mature forms of IL-18 and IL-1β and subsequent pyroptotic cell death (Zhu et al., 2018b). This cell death has been suggested to represent an innate immune response to cytosolic bacteria (Wallet et al., 2016, Zhu et al., 2018b). It has been demonstrated that the inflammasome and the Guanylate-binding proteins (GBPs) are essential, IFN-γ-inducible immune factors, that function mainly independently to control the *F*. *novicida* infection (Wallet et al., 2017). The inflammatory response resulting from an *F. novicida* infection is much accentuated in comparison to that resulting from infection with *F. tularensis* strains (Gillette et al., 2014; Wallet et al., 2017). Although macrophage infection with the LVS strain demonstrates less inflammation, the infection can be controlled upon IFN-γ-mediated activation (Edwards et al., 2010; Wallet et al., 2017), whereas the activation is insufficient to control the subspecies *tularensis* SCHU S4 strain (Wallet et al., 2017). Studies have identified multiple suppressive mechanisms utilized by the latter strain, including very rapid degradation of cytokine and chemokine mRNAs, and also metabolic reprogramming (Bauler et al., 2011; Bauler et al., 2014; Gillette et al., 2014; Wyatt et al., 2016; Jessop et al., 2018). These events coincide with the very rapid replication and dissemination within the host of SCHU S4 (Conlan et al., 2003).

Here, we characterized the responses of bone marrow-derived macrophages (BMDM) to infection with *F. tularensis* strains of variable virulence, the SCHU S4 strain, the LVS strain, or *F. novicida*. We observed that *F. novicida* infection resulted in a prominent proinflammatory response, whereas the response was much more muted upon infection with LVS and even more so with SCHU S4. Whereas, GBP expression was not affected by any of the *F. tularensis* strains, control of infection was only observed for *F. novicida* and LVS. In contrast, in cultures co-infected with *F. novicida* and SCHU S4, control of both bacterial strains was observed.

## Materials and Methods

### Bacterial Strains

*F. novicida* U112, *F. tularensis* LVS (subsp. *holarctica*), and *F. tularensis* strain SCHU S4 (subsp. *tularensis*) were obtained from the American Type Culture Collection and from the *Francisella* Strain Collection of the Swedish Defense Research Agency, Umeå, Sweden. Work with the SCHU S4 strain was performed in a biosafety level 3 facility certified by the Swedish Work Environment Authority.

### Animals

To obtain wild-type BMDM, C57/BL6 mice, obtained from Charles River, Germany, were used as a source. The source of GBP-deficient BMDM was *GBP^chr3–/–^* C57BL/6 mice, which have been previously described (Yamamoto et al., 2012). In the latter mice, the genes encoding GBP1, GBP2, GBP3, GBP5, GBP7, and GBP2PS are lacking, whereas expression of GBP4, GBP6, GBP8, GBP9, GBP10, and GBP11 is intact. Ethical approval for the described mouse experiments was obtained from the Ethical Committee on Animal Research, Umeå, Sweden, A67-14 and A36-2019 and University of Lyon, France (CEC-CAPP), protocol no. #ENS_2014_017 and #ENS_2017_002. The animal work was performed according to 2010/63/UE.

### Generation of BMDM

BMDM were prepared by collecting bone marrow from the femurs of mice and then plating the cells in Petri dishes in DMEM supplemented with 10% heat-inactivated fetal calf serum (FCS: Invitrogen Life Technologies), and 10% macrophage colony-stimulating factor (M-CSF)-conditioned medium. The latter was collected from the L929 cell line. After incubation at 37°C in 5% CO_2_ for 6 days, FCS-BMDM were harvested and added to 6-, 24-, or 96-well plates at the indicated density, and incubated overnight before infection. When applicable, BMDM were treated with 100 units/ml of mouse IFN-γ (#575306; Biolegend, San Diego, CA).

### Infection of BMDM

The *F. tularensis* strains were grown overnight on Gc-agar plates, then resuspended in cDMEM, and added to the BMDM monolayer at the indicated MOI. After uptake for 2 h, medium was removed and the macrophage monolayer was washed twice with 200 µl of FCS-DMEM, containing 20 µg/ml of gentamicin, was added to each well and plates were incubated for an additional 45 min and washed twice with FCS-DMEM. Bacterial counts were determined by lysis of the cells and plating of serial dilutions.

### Co-Infection Assay

A BMDM monolayer was infected with *F. novicida* at an MOI of 100 and with the same MOI of SCHU S4 or paraformaldehyde-killed SCHU S4 in the presence or absence of IFN-γ. After uptake for 2 h, the medium was removed and the macrophage monolayer was washed twice with 200 µl of FCS-DMEM containing 20 µg/ml of gentamicin and plates were incubated for an additional 45 min and washed twice. Bacterial counts were determined at indicated time points by lysis of the cells and plating of serial dilutions on Gc-agar plates. Discrimination of *F. novicida* and SCHU S4 was based on distinct colony morphology and growth kinetics. *F. novicida* colonies were greyish white and of sufficient size to count within 24 h, whereas SCHU S4 colonies were distinctly white and required 72 h of incubation before reaching sufficient size. Culture supernatants were also collected.

### Cytokine Analysis

Supernatants from cell cultures were collected, filtered by 0.22 μm Hydrophilic low protein binding Durapore membrane (Merck Millipore, Darmstadt, Germany) and stored at -80°C. Cytokine determinations were performed using the Bio-Plex 200 system with a multiplex kit #M60009RDPD (BioRad Laboratories, Hercules, CA, USA). Concentrations of IL-18, IL-1β, and IFN-β were measured by following the manufacturer’s protocols for the IL-18 ELISA kit (# KHC0181; Thermo Fisher Scientific, Waltham, MA), OptEIA mouse IL-1β ELISA kit (#559603**;**BD Biosciences, Franklin Lakes, NJ), and VeriKine Mouse IFN-b ELISA Kit #42400 PBL Assay Science, Piscataway, NJ, respectively.

### Western Blot Analysis

Wells were washed with ice-cold PBS and M-PER Reagent contains Halt Protease Inhibitor Cocktail (#78503; Thermo Scientific) was added to each well. The lysate was resuspended in 4×Laemmli sample buffer (#1610747; BioRad), and boiled at 95°C for 5 min before being analyzed by 8-16% polyacrylamide gel electrophoresis (#4561106; BioRad). Proteins transferred from gel to the 0.2 μm PVDF (#1704157; BioRad) by Turbo Transblot System (BioRad). Western blotting was performed with polyclonal rabbit anti-GBP2 (1:500; #11854-1-AP; Proteintech), anti-GBP5 (1:500; #13220-1-AP; Proteintech), or anti-IRGB10 serum (1:500; provided by Jonathan Howard, Instituto Gulbenkian de Ciencia, Oeiras, Portugal). The lysate was probed with monoclonal anti-GAPDH (1:1000; #AM4300; Thermo Fisher) as a loading control. The secondary antibody was conjugated to HRP (horseradish peroxidase). For quantitative analysis of protein bands, the Amersham imager 600 was used.

### Immunofluorescence

BMDMs were seeded on 8-well glass slides (Millicell EZ slide; #PEZGS0416) and were infected as described above. At the desired time points, cells were washed two times and fixed for 20 min at RT with 4% paraformaldehyde. Following fixation, cells were washed three times with PBST, and incubated with blocking buffer overnight at 4°C. Cells were stained for 1 h at RT with primary antibodies (identified below), then washed with PBST and incubated for 1 h at RT with the appropriate Alexa Fluor–conjugated secondary antibodies (identified below) (1:10,000 dilution; Invitrogen) were washed three times with PBST and 1X-DAPI (0.1μg/ml) was added to each well. After washing three times with PBST, mounting media (Invitrogen, #P36965) was added to each well and then coverslips added to the wells. Antibodies used were anti–*F. tularensis* LPS (1:1000 dilution; #MA1-21690; Invitrogen) to stain SCHU S4 and LVS, rabbit anti-GBP2 (1:500 dilution; 11854-1-AP; Proteintech) and anti-GBP5 (1:500 dilution; 13220-1-AP; Proteintech).

Coverslips were analyzed using Zeiss LSM710 Confocal and Leica Widefield Thunder microscopes at the magnification of ×63 or ×100, respectively. Total intracellular bacteria were quantified by an automated process using Fiji software (https://imagej.net/software/fiji/).

### Quantitative PCR

Total RNA was isolated using the TRIZOL Reagent (Invitrogen), quality and concentration of the RNA were determined by measuring A_260_ with a spectrophotometer (Nanodrop ND-1000) and then converted to cDNA by using the iScript cDNA synthesis kit (BioRad #1708890). qPCR was performed by using SsoAdvanced Universal SYBER Green PCR master mix (BioRad # 1725274) using forward and reverse primers from BioRad and Eurofins to quantify mRNA level of *gbp2*, *gbp5*, *ifi204, ligP*, and the β2-microglobulin gene *(b2m)*. The latter was amplified as an internal control. The unique primer sequences are listed in **Table S1** and the *ifi204* and *ligP* amplicons in **Table S2**. Amplification was performed by using QuantStudio5 with the following cycling parameters 95°C for 5 min, then 40 cycles of 95°C for 15 seconds, 60°C for 1 minute, and 95°C for 15 seconds, followed by a final extension at 60°C for 1 minute.

The Ct values of the selected genes were normalized using the *b2m* gene as a reference. The relative copy numbers were calculated using the equation RCN=2^-ΔCt^ (Gavrilin et al., 2006). ΔCt denotes the Ct of the indicated gene subtracted with the Ct of *b2m*.

### Data Analysis and Statistical Methods

Statistical analysis was performed by use of the GraphPad Prism Software 9.0.2. To analyze the significance of differences between different groups One-way ANOVA with Tukey’s correction for multiple analysis was performed. *P* values < 0.05 were considered significant.

## Results

### Intracellular Replication of *F. tularensis* Strains

**Figure 1:**
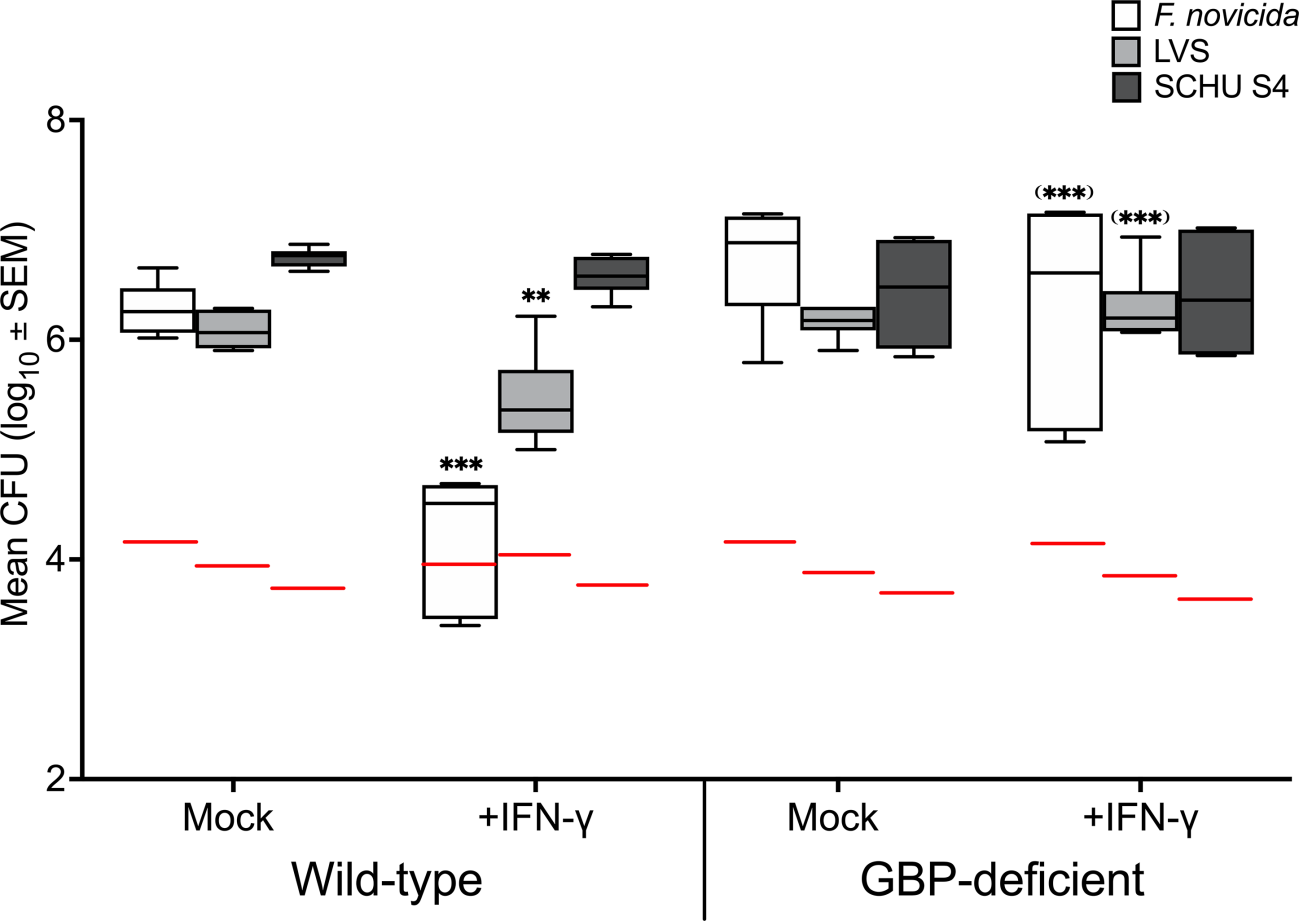
Intracellular proliferation of *F. tularensis* strains in wild-type BMDM and GBP^-/-^ BMDM. BMDM monolayer was infected using an MOI of 100 with SCHU S4, LVS or *F. novicida* in IFN-γ primed and non-primed cultures. CFU numbers were recorded from triplicate wells and data are representative of three independent experiments, In each box plot, the line through each box shows the median, with quartile one and three as the lower and upper limits of each box. The end of the vertical lines indicates maximum and minimum values, respectively. The significances indicated without parenthesis are in comparison to mock wild-type BMDM and those within parenthesis indicate the values in comparison to the same infection of wild-type BMDM. **P < 0.01, ***P < 0.001. The red lines indicate the inocula.

To assess the ability of *F. tularensis* strains to replicate intracellularly, BMDM were infected with *F. novicida*, LVS, or SCHU S4. Each of the strains demonstrated effective intracellular replication; in wild-type BMDM an increase of 2.0 - 2.5 log_10_ CFU after 20 h (**Figure 1**). In the presence of IFN-γ, the increase was significantly lower for LVS and *F. novicida*, 1.2 log_10_ CFU and 0.2 log_10_ CFU (*P <* 0.01 and 0.001, respectively), respectively, whereas the number of SCHU S4 was not affected by IFN-γ; a net increase of 2.0 log_10_ CFU was observed (**Figure 1**; *P* > 0.05). In GBP-deficient BMDM, the number of CFU increased between 2.0 and 2.6 log_10_, whereas the addition of IFN-γ led to no change in the number of LVS or SCHU S4 (**Figure 1**). The number of *F. novicida* showed a non-significant decrease of 0.4 log_10_ CFU (**Figure 1**). When compared to the wild-type cultures, the number of LVS and *F. novicida* bacteria were significantly higher in the cultures with GBP-deficient BMDM, *P <* 0.001, whereas there was no significant difference for SCHU S4, *P* > 0.05 (**Figure 1**).

The data demonstrate that addition of IFN-γ confers effective GBP-dependent control of LVS and *F. novicida*, whereas it confers no control of the SCHU S4 infection. In the absence of GBPs, no significant control of any infection occurred. The findings are in agreement with previous results based on the three strains (Wallet et al., 2017).

### Regulation of GBPs During *F. tularensis* Infection

In view of the important role of GBP for the control of the LVS and *F. novicida* infections, but not the SCHU S4 infection, it was investigated whether expression of GBP2 and GBP5 was differentially affected at the gene or protein levels by the infections.

**Figure 2:**
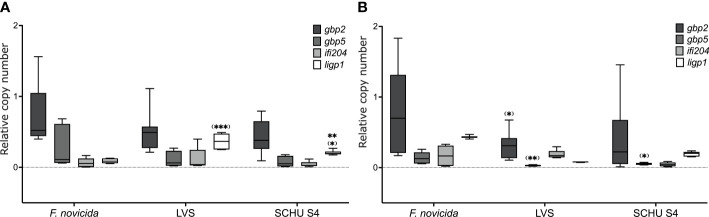
RT-PCR analysis of *gbp2*, *gbp5*, *ifi204* and *ligP* expression in cultures with wild-type BMDMs after addition of IFN-γ and infected with indicated *F. tularensis* strains using an MOI of 100. The box plots represent relative copy number of mentioned genes 4 h **(A)** and 8 h **(B)**. Significances within parenthesis indicate comparisons to *F novicida*-infected BMDMs. *P < 0.05, **P < 0.01, ***P < 0.001.

To analyze whether the *F. tularensis* infections affected the regulation of IFN-γ-inducible genes, the transcription of the *GBP2* and *GBP5* genes and two other well-known IFN-γ-inducible genes, *ifi204* and *ligP* (Storek et al., 2015), were analyzed by RT-PCR. Expression was normalized by measurements of levels of *b2m.* Expression of *GBP2* and *GBP5* was very similar and not significantly different between uninfected cells and cells infected with either of the three *F. tularensis* strains at the 4 h time point, whereas at the 8 h time point, the level of *GBP2* was higher in *F. novicida*-infected BMDM compared to LVS-infected BMDM and *GBP5* was higher in *F. novicida*-infected BMDM compared to either of the other infected BMDM (**Figures 2A, B**). Levels of *ligP* were significantly lower in *F. novicida*-infected BMDM compared to LVS or SCHU S4-infected BMDM and higher in LVS-infected BMDM *vs.* SCHU S4-infected BMDM at the 4 h time point. There were no significant differences between the uninfected and infected cell cultures with regard to the IFN-γ-inducible gene, *ifi204* at the 4 h or the 8 h time point (**Figures 2A, B**).

**Figure 3:**
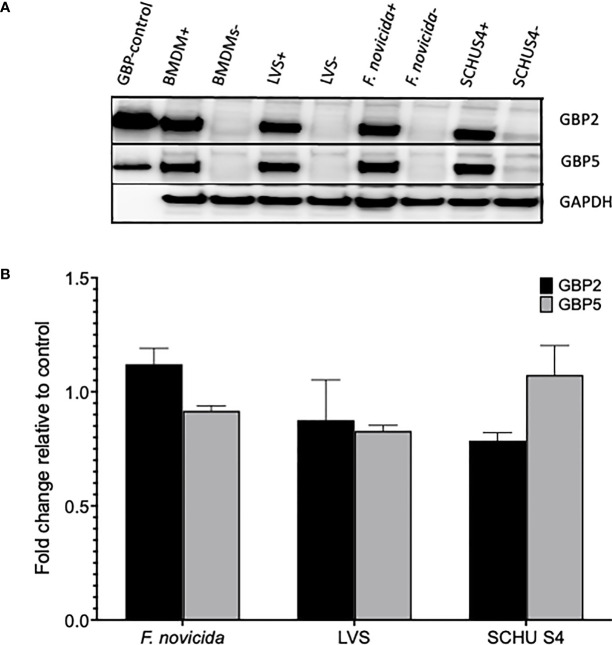
**(A)** Western blot analysis of GBP2, GBP5, and GAPDH expression in cultures with wild-type BMDMs in the presence of (indicated with +), or absence of IFN-γ (indicated with -) were infected with indicated *F tularensis* strains using an MOI of 100. **(B)** Quantification by densitometry of **(A)**. Mean values and SEM are indicated.

The protein expression in cell cultures with or without addition of IFN-γ was evaluated using Western blot analysis after 20 h of infection. As controls, expression of GAPDH and IRGB10, the latter an IFN-γ-inducible protein was analyzed. Although there were certain differences between the expression of the GBP proteins depending on the infectious agents and between experiments, they were minor and non-significant (**Figure 3**). Likewise, expression of IRGB10 was not affected by the infections, neither in wild-type cell cultures, nor in cultures with GBP-deficient cells (not shown).

We conclude that transcription of IFN-γ-inducible genes was somewhat affected by infection with either of the *F. tularensis* strains at the 8 h time point, but not at the 4 h time point. Regardless of these transcriptional differences, the IFN-γ-induced expression of GBP2 and GBP5 was not affected by the infections.

### Expression of GBP2 and GBP5 in *F. tularensis*-Infected BMDM

**Figure 4:**
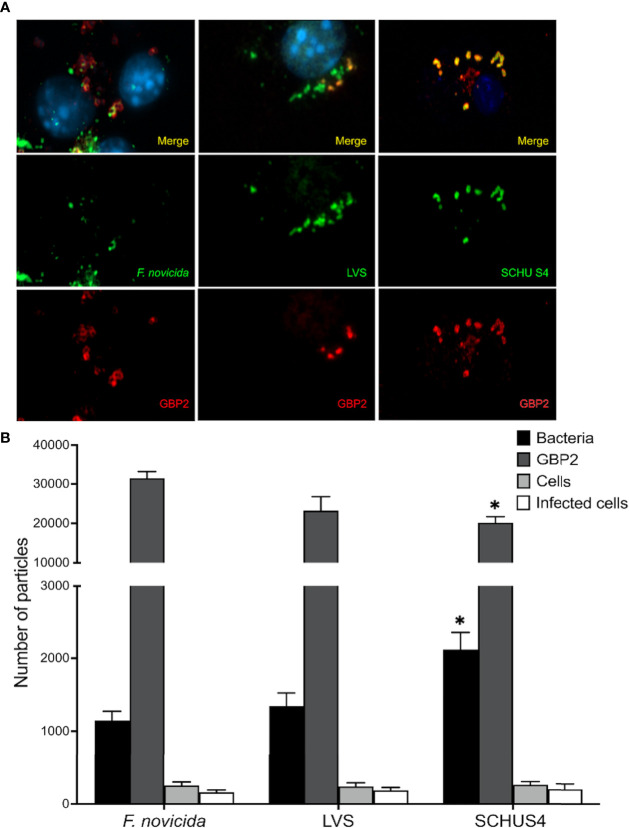
Immunofluorescence staining of *F tularensis* (green), GBP2 (red), and DNA (blue) in IFN-γ-treated BMDMs infected (MOI = 500) for 8 h with *F novicida*, LVS, or SCHU S4 **(A)**. Results for a representative experiment are shown in **(B)**. *P < 0.05 *vs. F novicida*-infected cultures.

To further analyze if the expression of GBP2 and GBP5 was affected in cell cultures infected with *F. tularensis*, expression at the cellular level was analyzed with confocal microscopy. BMDMs were infected with either *F. novicida*, LVS, or SCHU S4 and the number of bacteria and the degree of GBP expression per cell was quantified at 4 h and 8 h after start of infection. The findings were similar at the different time points so therefore, the 8 h time point was used in repeated experiments. Micrographs illustrating each infection is shown in **Figure 4A**. The number of bacteria per cell, GBP2 expression, and the number of infected cells were not significantly different in cultures infected with *F. novicida* and LVS, whereas the SCHU S4-infected cultures, in comparison to the *F. novicida*-infected cultures, contained higher number of bacteria (*P* < 0.05) and lower number of GBP2 (*P* < 0.05) (**Figure 4B**). Co-localization between GBP2 and bacteria was observed (**Figure 4A** and **Figures S1A–C**) and there were 0.51 co-localized *F. novicida* per cell; whereas the numbers were significantly lower (*P* < 0.001) for LVS and SCHU S4, 0.12 and 0.08, respectively.

### Production of IL-1β and IL-18 Upon *F. tularensis* Infection

*F. novicida* has served as a prototype for activation of the AIM2 inflammasome and it has been demonstrated that the infection leads to activation of caspase-1 and cleavage of the precursors of IL-1β and IL-18, thereby leading to their secretion (Fernandes-Alnemri et al., 2010).

**Figure 5:**
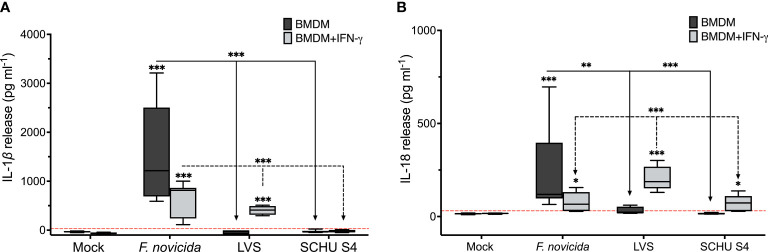
Secretion of IL-1β **(A)** and IL-18 **(B)** from BMDM in the presence or absence of IFN-γ upon infection with indicated *F tularensis* strain using an MOI of 100 after 18 h of infection. Data represent median ± SEM from three independent experiments. **P < 0.01, ***P < 0.001. The red dotted line indicates the limit of detection.

Accordingly, we observed high levels of secreted IL-1β and IL-18 in *F. novicida*–infected cultures (**Figures 5A, B**). Addition of IFN-γ resulted in decreased levels of secreted cytokines, although they still were very significant. Upon infection with the LVS strain, levels of both cytokines were around the limit of detection in untreated BMDM, whereas they were significantly higher in cultures with wild-type BMDM in the presence of IFN-γ (**Figures 5A, B**). In contrast, infection of wild-type BMDM with SCHU S4 resulted in no measurable levels of IL-1β and in the presence of IFN-γ, low levels of IL-18, whereas no IL-18 was secreted in the absence of IFN-γ.

Thus, each of the three strains induced distinct patterns of cytokine release and the low or no levels observed during the SCHU S4 infection were notably different from the patterns observed with the other two strains.

### Intracellular Replication of *F. novicida* and SCHU S4 in a Co-Infection Model

**Figure 6:**
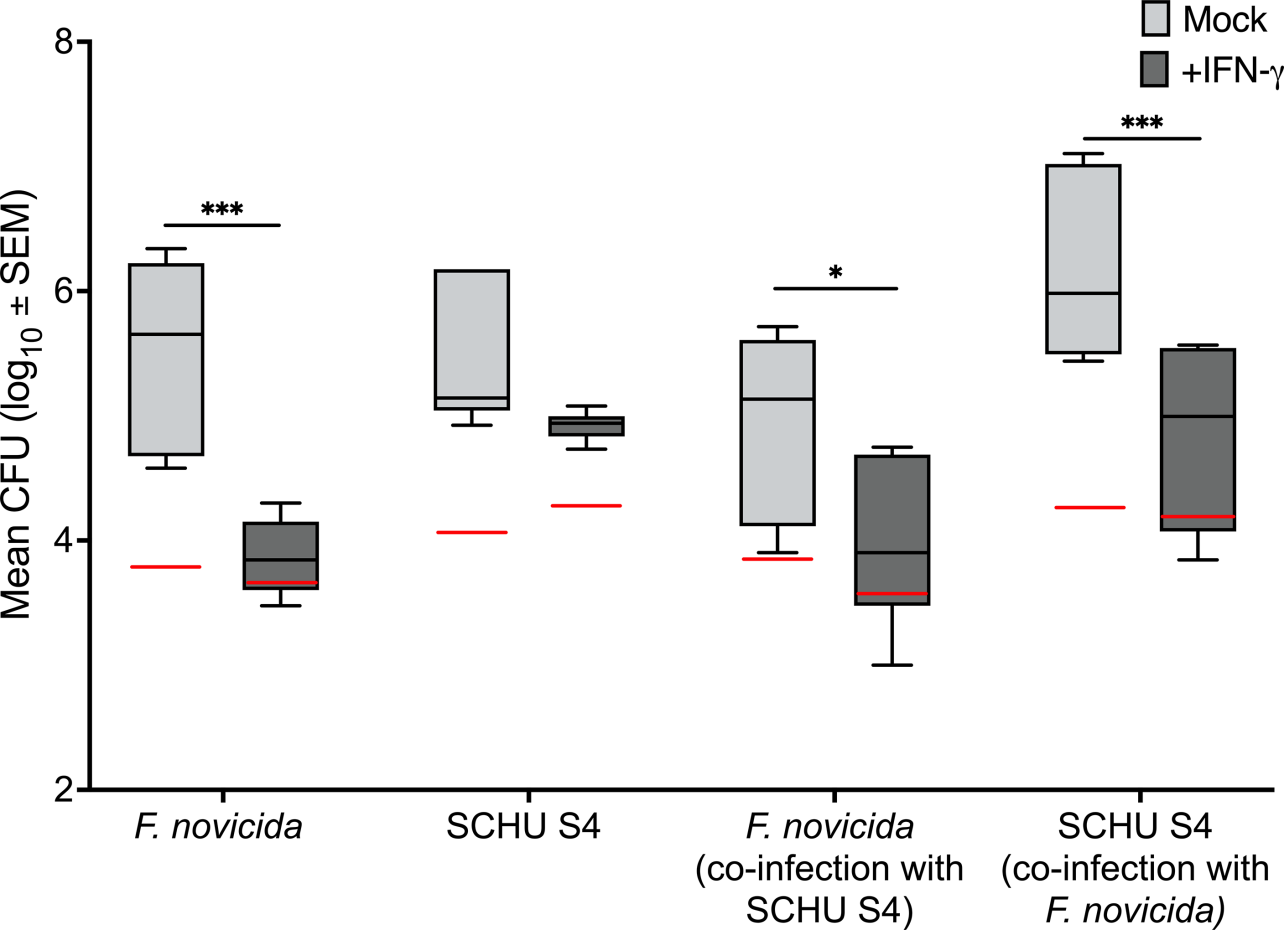
Intracellular replication of *F. novicida* and SCHU S4 in a co-infection model. BMDM were infected with either *F. novicida* or SCHU S4 using an MOI of 100, or both strains in the presence or absence of IFN-γ. *P < 0.05, ***P < 0.001. Red lines indicate the inocula.

In view of the findings summarized in **Figure 1**, demonstrating that IFN-γ treatment of *F. novicida*-infected BMDM resulted in prominent control of infection, we asked whether there would be control of the SCHU S4 infection upon co-infection with *F. novicida*. Therefore, BMDM were infected with either *F. novicida* or SCHU S4, or both strains in the presence or absence of IFN-γ. As previously observed, addition of IFN-γ resulted in very prominent control of the *F. novicida* infection, *P <* 0.001, whereas no control of the SCHU S4 infection occurred, *P* > 0.05 (**Figure 6**). During co-infection, again, significant control, *P <* 0.05, of the *F. novicida* infection was observed, but also the number of SCHU S4 bacteria were significantly lower with IFN-γ treatment, *P <* 0.001 (**Figure 6**).

Thus, unlike the lack of control observed when IFN-γ-treated BMDM were infected with SCHU S4, there was significant control during co-infection, implying that intracellular mechanisms not active during infection with SCHU S4 alone, could be activated during the co-infection.

### Cytokine Secretion in the Co-Infection Model

To better understand the mechanisms underlying the control of the SCHU S4 infection in the co-infection model, multiplex cytokine analysis was performed from the supernatants after addition of IFN-γ. In total, 18 cytokines were analyzed and there were marked differences between the cultures with *F. novicida* and SCHU S4 in as much as levels of almost all cytokines were much lower in the latter cultures (**Figure S2**). Out of the 17 cytokines, 15 were significantly lower in the SCHU S4 cultures and the only exceptions were IL-13 and MIP-1α, but the level of the latter cytokine was very low in the SCHU S4 cultures (**Figure S2**). For most cytokines, levels in the co-infection cultures were intermediary compared to the levels in the cultures infected with *F. novicida* or SCHU S4 and 10 cytokines were significantly higher in the co-cultures than in the SCHU S4-infected cultures, whereas 8 cytokines were significantly lower in the co-infection cultures compared to the *F. novicida*-infected cultures (**Figure S2**). As control, also killed SCHU S4 bacteria were added to *F. novicida*-infected cultures and all cytokine levels were at least as high as those in the cultures with *F. novicida* alone.

Thus, the proinflammatory properties of *F. novicida* in the co-infection model resulted in high or intermediate levels of most of the 18 cytokines, in contrast to the very low levels observed in the SCHU S4-infected cultures. The low levels were dependent on the presence of viable SCHU S4 bacteria, since addition of killed bacteria generally resulted in very high cytokine levels. Co-infection resulted in cytokine levels mostly intermediate to the levels observed with either pathogen alone.

## Discussion

For more than three decades, the unique role and many facets of the IFN-γ-mediated mechanisms for protection against intracellular bacteria have been investigated and subsequently become better and better understood (Bryson, 2021). The cytokine induces hundreds of genes in various host defense pathways and with regard to *F. tularensis*, it has been observed that IFN-γ is critically required, although alone not sufficient for host protection (Edwards et al., 2010; Nallaparaju et al., 2011; Wallet et al., 2017). For long, it has been elusive how the IFN-γ-induced bactericidal effects are executed and many potential mechanisms have been excluded, *e.g*., cell death, autophagy, production of reactive oxygen or nitrogen species, tryptophan degradation, or activation of caspase-1 and caspase-11 (Edwards et al., 2010; Nallaparaju et al., 2011). Rather recently, the critical role of GBPs for execution of the IFN-γ-induced control of *F. tularensis* infection was identified (Man et al., 2015; Meunier et al., 2015). In fact, studies on *F*. *novicida* and LVS have indicated that all bactericidal mechanisms in BMDM were exclusively dependent on GBPs, whereas the IFN-γ-mediated antibacterial control of SCHU S4 infection was found to be marginal (Wallet et al., 2017). Moreover, in a model of acquired immunity, it was found that control of infection was GBP-dependent, irrespective of the virulence of the infecting strain (Mohammadi et al., 2020). In addition, besides the specific roles for GBPs in controlling an *F. tularensis* infection, there is accumulating evidence demonstrating their crucial roles in controlling numerous other intracellular infections (Tretina et al., 2019).

One important GBP-mediated antibacterial mechanism is to serve as platform for the assembly of inflammasome complexes, a prototypic example is the AIM2 inflammasome activation during the *F. novicida* infection (Fernandes-Alnemri et al., 2010; Man et al., 2015; Meunier et al., 2015). During the infection, it has been demonstrated that the presence of GBPs lead to cytosolic lysis of *F. novicida* and the released DNA may serve as a scaffold for AIM2 oligomerization (Man et al., 2015; Meunier et al., 2015). Moreover, GBPs have been suggested to serve as master regulators of numerous inflammasomes during the *F. novicida* infection (Wallet et al., 2017). Despite the central role of GBPs in this regard, it has been demonstrated that control of intracellular replication occurs independently of inflammasomes, but strictly dependent on GBPs (Wallet et al., 2017). For other Gram-negative bacteria, it has been demonstrated that GBPs lead to control of infection by both direct and indirect means, including bacterial lysis by destabilizing the rigidity of the bacterial outer membrane, due to LPS clustering (Kutsch et al., 2020). There appears to be principal differences between the *F. novicida* infection and the LVS and SCHU S4 infections. One notable difference is that SCHU S4, unlike *F. novicida*, not only activates the AIM2 inflammasome in murine BMDM, but also the NLRP3 inflammasome and, in fact, it has been demonstrated that the latter was mandatory for release of IL-1β, whereas IL-18 secretion was dependent on both inflammasomes (Atianand et al., 2011). We observed that infection with each strain induced a unique pattern of release of IL-1β and IL-18, implying that their engagements of inflammasomes are distinct. These findings are in agreement with previously published data (Atianand et al., 2011; Wallet et al., 2017).

Western blot analysis of GBP expression demonstrated that the each of three *F. tularensis* strains did not significantly affect translation in IFN-γ-treated cells. GBP expression in the infected cells was also analyzed at the cellular level by microscopy and again, there were no significant differences. Despite these similarities, there was a striking difference between the *F. tularensis* strains with regard to their susceptibility to the IFN-γ treatment. Whereas the effect was very marked in cultures infected with LVS or *F. novicida*, there was no significant control in cultures infected with SCHU S4. The findings are in agreement with previously published data (Wallet et al., 2017). Moreover, our findings demonstrate that the IFN-γ-mediated control of the *F*. *novicida* and LVS infections is exclusively dependent on the presence of GBPs, since addition of IFN-γ did not lead to any significant control of infection in cultures with GBP-deficient BMDM. Altogether, the findings indicate that the SCHU S4 strain has evolved mechanism(s) to avoid the IFN-γ-mediated GBPs-dependent antibacterial activity. Notably, despite this avoidance, as aforementioned, overall expression of GBP2 and GBP5 was not dependent on the infecting strains. Thus, this demonstrates that innate immune sensors recognize cytosolic SCHU S4 bacteria and therefore implies that the strain actively avoids the anti-bacterial mechanism. In support, there are a number of previous publications demonstrating that SCHU S4 actively suppresses numerous anti-bacterial mechanisms (Bauler et al., 2011; Bauler et al., 2014; Gillette et al., 2014; Wyatt et al., 2016; Jessop et al., 2018).

We hypothesized that the prominent inflammatory responses apparent during the *F. novicida* may endow the host cells with an ability to control even the SCHU S4 infection. Indeed, upon co-infection with *F. novicida* and SCHU S4, significant control of both strains was observed. This implies that intracellular mechanisms not active during infection with SCHU S4 alone, possibly inhibited by an active bacterial mechanism, could be activated during the co-infection. Corroborating the hypothesis, we observed that addition of killed SCHU S4 bacteria to *F. novicida*-infected cultures resulted in the secretion of high levels of all cytokines. The hypothesis was further supported by very distinct patterns of secreted cytokines from the cultures infected with SCHU S4 or *F. novicida* vs. the co-infection pattern. Whereas levels in the SCHU S4-infected cultures were very low, or not measurable for almost all cytokines, levels in the co-infected cultures were intermediate or not significantly different from the high levels of cytokines observed in the *F. novicida*-infected cultures. Thus, the pro-inflammatory properties of *F. novicida* was sufficient to overcome the anti-inflammatory properties executed by SCHU S4. A co-infection protocol has been used in a previous study and in agreement with the present findings, it was observed that addition of SCHU S4 suppressed the pro-inflammatory cytokine response to an existing *F. novicida* infection (Gillette et al., 2014).

Collectively, our findings demonstrate that *F. tularensis* strains exhibit unique characteristics during infection of BMDM, as evidenced by cytokine patterns and susceptibility to GBP-dependent infection control; however, these differences are not due to manipulation of GBP expression in infected cells. Whereas strains of low virulence are highly susceptible to GBP-dependent control, the highly virulent SCHU S4 strain is not, demonstrating that this characteristic correlates to virulence.

## Data Availability Statement

The original contributions presented in the study are included in the article/**Supplementary Material**. Further inquiries can be directed to the corresponding author.

## Ethics Statement

The animal study was reviewed and approved by the Ethical Committee on Animal Research, Umeå, Sweden and the University of Lyon, France (CEC-CAPP).

## Author Contributions

NM, and HL performed the experiments. AM prepared GBP chr3-deficient macrophages. AS, NM, and HL designed the study, analyzed the data, and wrote the manuscript. TH wrote and reviewed the manuscript. MY provided the GBP chr3-deficient mice. All authors contributed to the article and approved the submitted version.

## Funding

We acknowledge research funding for this work by grants to AS from the Swedish Research Council, Medicine and Health, 2020-01362 and Region Västerbotten, Centrala ALF-medel, RV-939171, Basenhets-ALF, RV-941049, and Spjutspetsmedel.TH received funding from the FINOVI foundation and NB from the Kempe foundation.

## Conflict of Interest

The authors declare that the research was conducted in the absence of any commercial or financial relationships that could be construed as a potential conflict of interest.

## Publisher’s Note

All claims expressed in this article are solely those of the authors and do not necessarily represent those of their affiliated organizations, or those of the publisher, the editors and the reviewers. Any product that may be evaluated in this article, or claim that may be made by its manufacturer, is not guaranteed or endorsed by the publisher.
