# Supplementary material for: Macrophages Demonstrate Guanylate-Binding Protein-Dependent and Bacterial Strain-Dependent Responses to Francisella tularensis
Source: Front Cell Infect Microbiol. 2021 Dec 24;11:784101. doi: 10.3389/fcimb.2021.784101 (PMC8738097; doi:10.3389/fcimb.2021.784101)
Supplement: Supplementary file 1 [file DataSheet_1.pdf]

Fig. S1A

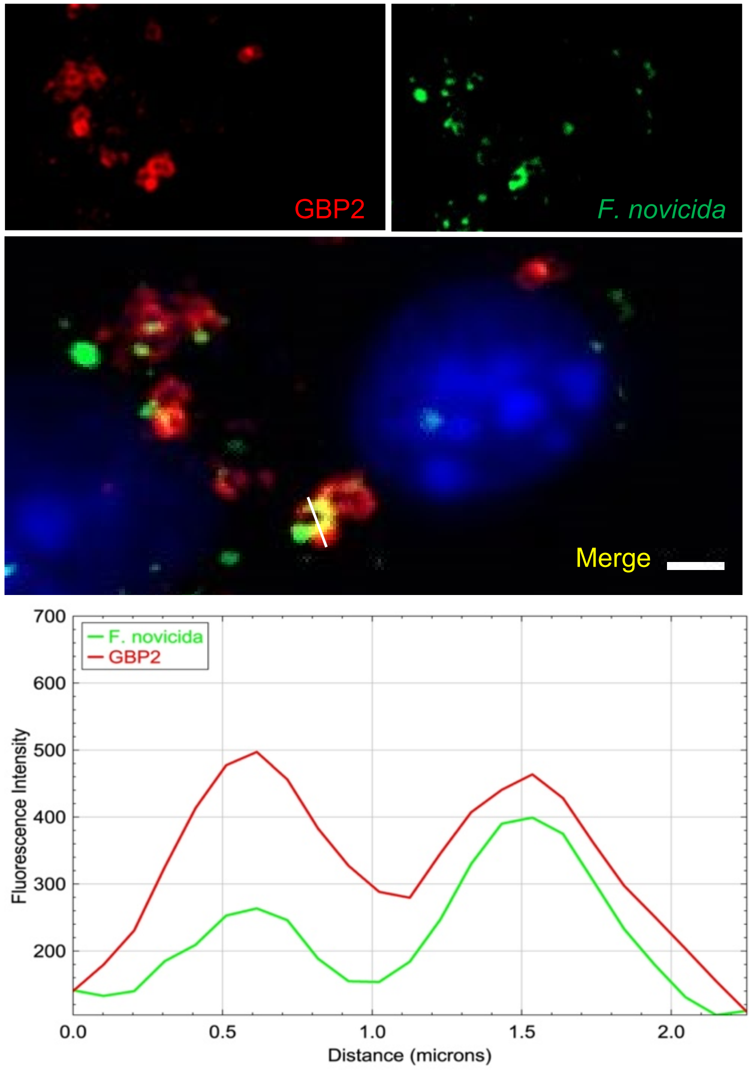

GBP2
F. novicida
Merge
700
600
500
400
300
200
Fluorescence Intensity
F. novicida
GBP2
0.0
0.5
1.0
1.5
2.0
Distance (microns)

Fig. S1B

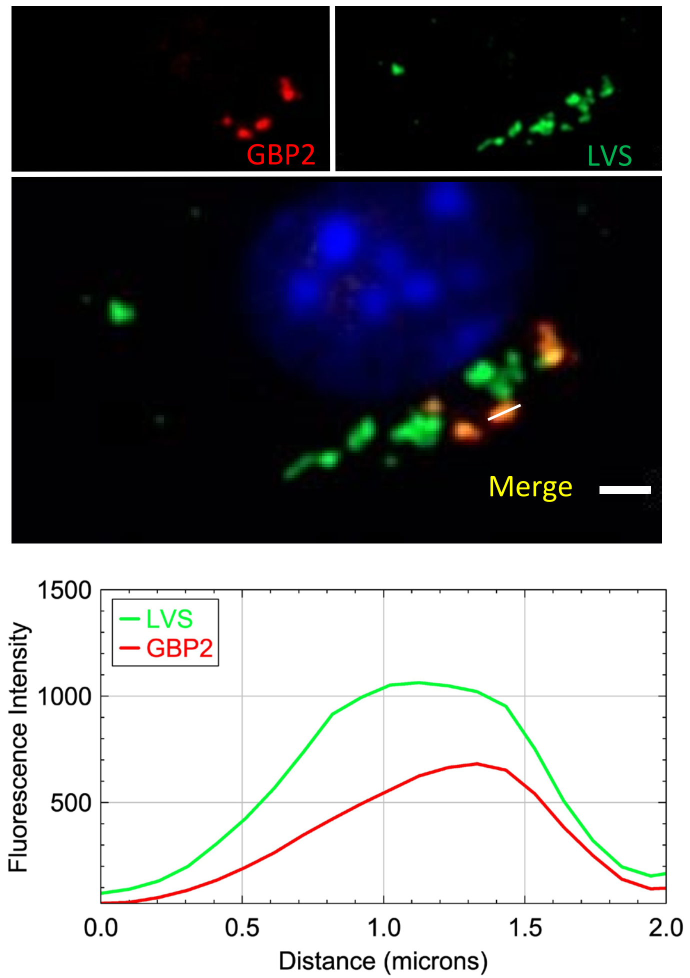

GBP2
LVS
Merge
1500
1000
500
0.0
0.5
1.0
1.5
2.0
LVS
GBP2
Fluorescence Intensity
Distance (microns)

Fig. S1C

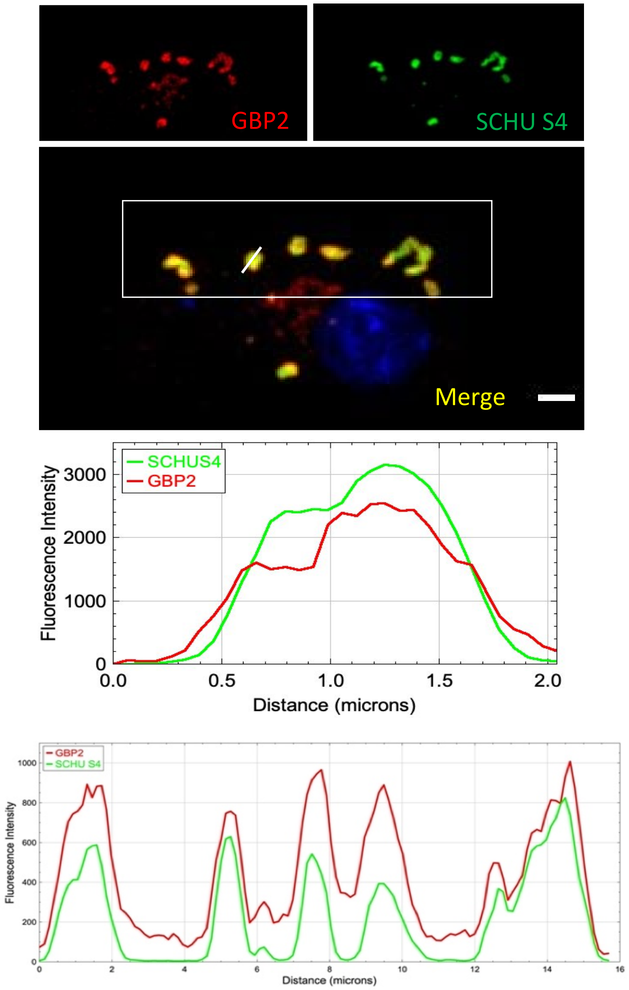
GBP2
SCHU S4
Merge
SCHUS4
GBP2
Fluorescence Intensity
3000
2000
1000
0
0.0
0.5
1.0
1.5
2.0
Distance (microns)
GBP2
SCHU S4
Fluorescence Intensity
Distance (microns)

Fig. S1. Co-localization of indicated *Francisella* strains and GBP2 in infected BMDMs. Graphs represent fluorescence intensity profiles from samples co-immunostained for indicated *Francisella* strain (green channel) and GBP2 (red channel) 8 h post-infection. (A) *F. novicida* and GBP2, (B) LVS and GBP2, and (C) SCHU S4 and GBP2. The line profile plots under each image indicate the intensity distribution of green and red channels through the white lines or box in the merged panel. The fluorescence intensities are plotted along the y-axis. Scale bar is 2.0 µm.

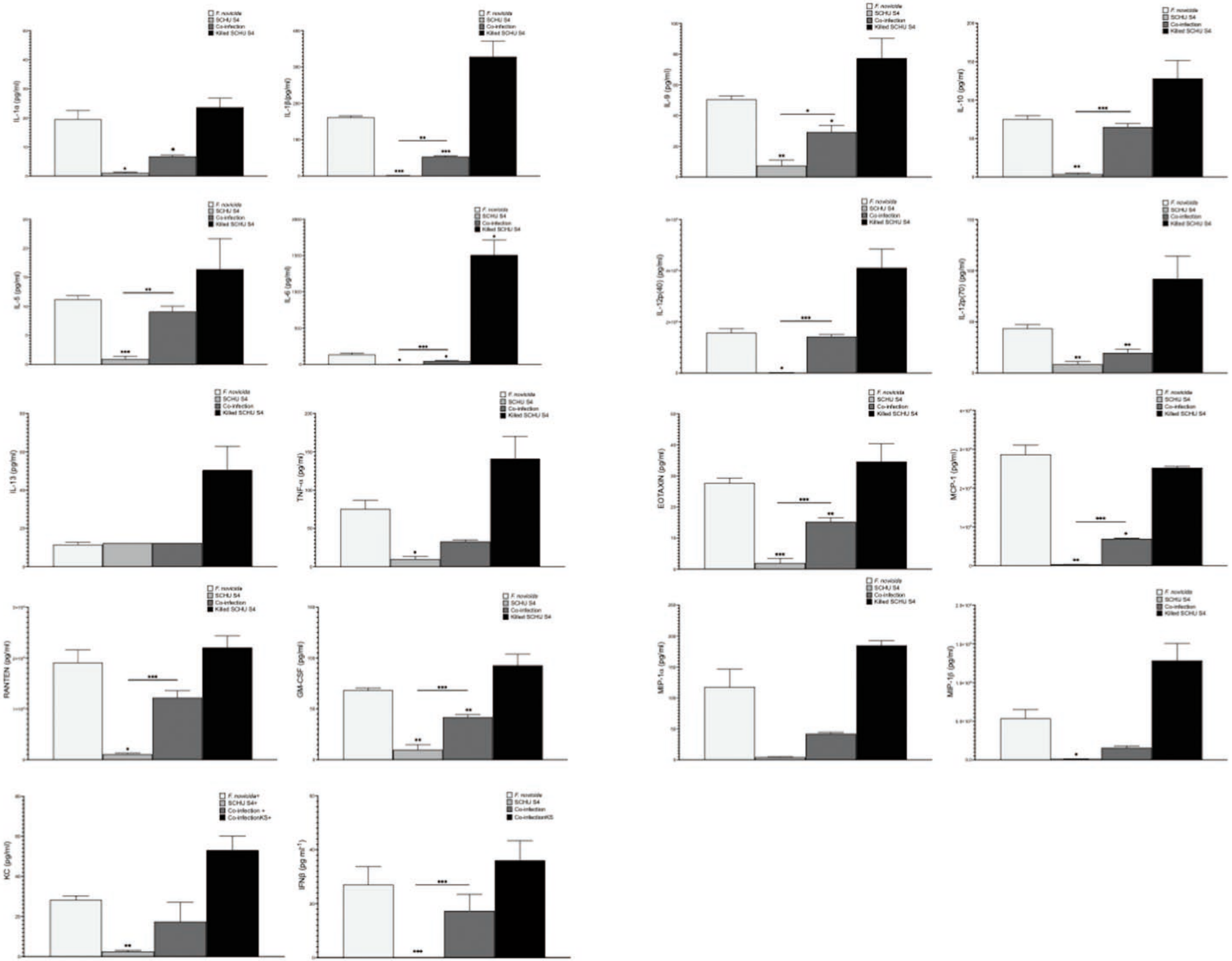
F. novicida
SCHU S4
Co-infection
Killed SCHU S4
IL-1α (pg/ml)
IL-1β (pg/ml)
IL-9 (pg/ml)
IL-10 (pg/ml)
IL-5 (pg/ml)
IL-6 (pg/ml)
IL-12p(40) (pg/ml)
IL-12p(70) (pg/ml)
IL-13 (pg/ml)
TNF-α (pg/ml)
EOTAXIN (pg/ml)
MCP-1 (pg/ml)
RANTEN (pg/ml)
GM-CSF (pg/ml)
MIP-1α (pg/ml)
MIP-1β (pg/ml)
KC (pg/ml)
F. novicida+
SCHU S4+
Co-infection +
Co-infectionKS+
IFNβ (pg ml⁻¹)
Co-infectionKS

Fig. S2. Secretion patterns of 18 cytokines in the co-infection assay. Supernatants were collected 18 h post-infection of cultures with IFN-γ-treated BMDMs infected with the indicated bacterial strain and subjected to cytokine analysis. The data are from three independent experiments and the bars indicate the mean ± SEM.

Table S1. List of primer sequences used for RT-PCR analysis. Primers were designed by online software https://eurofinsgenomics.eu/en/ecom/tools/pcr-primer-design/

Gene symbol	Forward 5´->3´	Reverse 5´->3´
b2m	tgaccggcttgtatgctatc	cagtgtgagccaggatatag
gbp2	accagctgcactatgtgacg	tcagaagtgacgggttttcc
gbp5	ccagagtaaagcggaacaag	gtgcaactcttgccttctcc

Table S2. Amplicons analyzed by the RT-PCR analysis for *ligp1* and *ifi204*. Corresponding primers were purchased from BioRad.

Gene	UniGene ID	Ensembl ID	Amplicon Sequence	Amplicon Length (bp)
ligp1	Mm.261140	ENSMUSG00000054072	GGGCCAGCTATCCTGCCACATGCAGTAGTAAATAAG AGGCCCTGACTCAAGCAAGTGGAAGGTAAAAGATAA CAAGCAAGCTCATCCTTTGATCTCTGCATACATGC	77
ifi204	Mm.261270	ENSMUSG0000007348	GTGTTCTCCACACACTAACTTTGGTTGCCTGTCAATT GTTTTCAGGTGAAAGCAGAAGAGGTGGAGTTTATCTC CTTCCTTGCAGTTGATGTTGTGCCATTTTCCACTCCCC ACCACTTCTATGCTTCCTGAACCATCTTTTATTTCATA GATTGTGTTCTTTCGGTTCACTGTTTTCTTGTGTAATG TAAACACTCCATAGAACACTGCTCCAGAAGTACC CT	194
